# Nano-Raman Spectroscopy Figure of Merit and Chemical Analysis of Contaminations in Single-Layer MoSe_2_

**DOI:** 10.1021/acsnano.5c08036

**Published:** 2025-09-11

**Authors:** Jane Elisa Guimarães, Rafael Nadas, Rayan Alves, Wenjin Zhang, Takahiko Endo, Kenji Watanabe, Takashi Taniguchi, Riichiro Saito, Yasumitsu Miyata, Bernardo R. A. Neves, Ado Jorio

**Affiliations:** † Departamento de Física, Universidade Federal de Minas Gerais, Belo Horizonte, Minas Gerais 31270-901, Brazil; ‡ Institut fur Physik, Humboldt-Universitat zu Berlin, Newtonstraße 15, 12489 Berlin, Germany; § Department of Physics, National Taiwan Normal University, Taipei 106, Taiwan; ∥ Research Center for Materials Nanoarchitectonics, National Institute for Materials Science, Tsukuba 305-0044, Japan; ⊥ Research Center for Electronic and Optical Materials, National Institute for Materials Science, Tsukuba 05-0044, Japan; # Department of Physics, Tohoku University, Sendai 980-8578, Japan; △ Department of Physics, Tokyo Metropolitan University, Tokyo 192-0397, Japan

**Keywords:** nano-Raman spectroscopy, tip-enhanced Raman spectroscopy, two-dimensional materials, nanoprotuberances, heterostructures, transition metal dichalcogenides, MoSe_2_

## Abstract

Contaminations in the formation of two-dimensional heterostructures can hinder or generate the desired properties. Recent advancements have highlighted the potential of tip-enhanced Raman spectroscopy (TERS) for studying materials in the 2D semiconductor class. In this work, we investigate the influence of 50–200 nm sized nanoprotuberances within a monolayer of MoSe_2_ using nano-Raman spectroscopy, establishing correlations between the presence of localized contaminations and the observed hyperspectral variations. A figure of merit is established for the identification of surface impurities, based on the MoSe_2_ peaks ratio resulting from TERS field coherence. New spectral peaks are also identified, which are associated with the presence of nanoprotuberances and indicate contamination and oxidation with localized charge transfer between MoSe_2_ and contaminant species.

## Graphical abstract

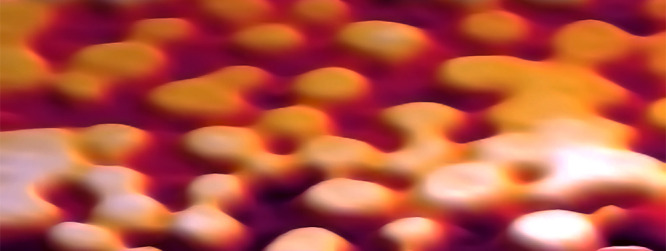

## Introduction

Transition-metal dichalcogenides (TMDCs), such as MoSe_2_, are increasingly studied due to their semiconducting properties and potential in optoelectronic applications.^1,2^ These materials are particularly valuable for enhancing device performance, making it essential to investigate their vibrational behaviors,^3,4^ excitonic interactions,^5,6^ and strain effects.^7^

Van der Waals heterostructures, created by stacking thin atomic layers of different materials, enable the combination of materials with complementary properties.^8^ Although these structures foster strong adhesion between adjacent two-dimensional materials, contamination can be present on the surfaces of each layer prior to assembly.^9^ This contamination, which may include adsorbed water molecules, oxidation, or hydrocarbons, is usually confined to nanoscale protuberances.^10,11^ When these protuberances are formed by trapped gases or droplets, they are commonly called nanobubbles.^12,13^

Tip-enhanced Raman spectroscopy (TERS) is a material characterization technique that provides nanometric resolution for nano-Raman spectroscopy.^14–16^ The maximum spatial resolution achievable with conventional optical microscopy is approximately half of the excitation wavelength. TERS was developed to overcome this limitation^17–19^ by combining scanning probe microscopy (SPM) with Raman spectroscopy. By employing a metallic tip to locally amplify the electromagnetic field, the technique provides enhancement of the Raman signal and improvement of spatial resolution, enabling detailed characterization of materials at the nanometer scale.^20^

In this study, nano-Raman hyperspectral measurements have been used to investigate nanoscale protuberances on a MoSe_2_ monolayer (Figure 1). These protuberances can induce localized variations in strain, dielectric environment, and charge distribution, potentially altering the vibrational modes and optical responses of surfaces.^21,22^ Here, we define a figure of merit for surface contamination based on the properties of MoSe_2_ peaks. Additionally, new peaks, unrelated to the intrinsic properties of the TMDCs, are here shown to emerge due to trapped contaminants within nanoprotuberances.

**Figure 1:**
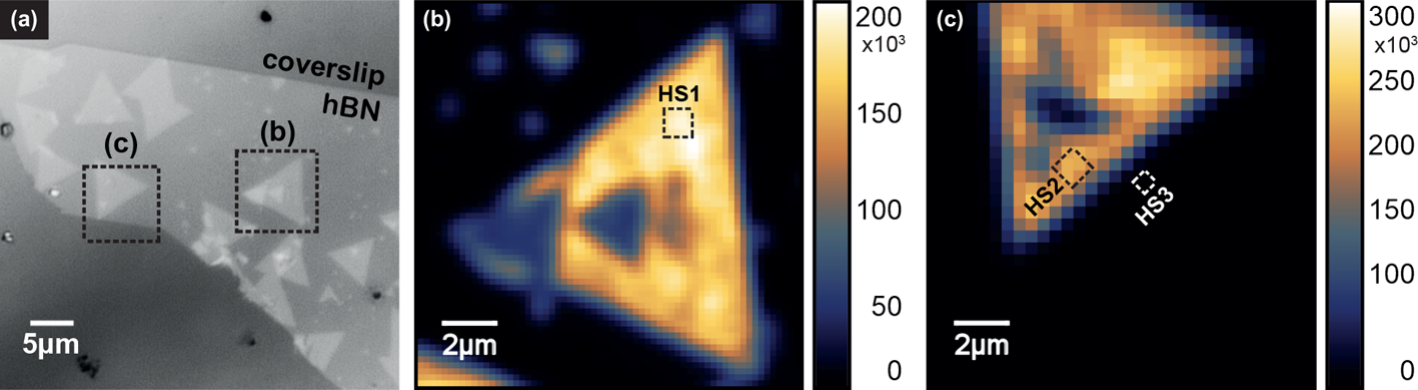
(a) Optical microscopy image of the MoSe_2_/hBN sample, showing regions of the monolayer and bilayer over an hBN flake. The highlighted triangles correspond to the areas where different nano-Raman hyperspectra were measured, indicated in (b, c). (b) Confocal photoluminescence (PL) intensity map acquired with an avalanche photodiode (APD) system over the MoSe_2_ heterostructure region where the HS1 nano-Raman hyperspectrum was collected. (c) PL intensity map acquired with the same APD system over the region where the HS2 and HS3 nano-Raman hyperspectra were collected. The color scale bars in (b,c) represent the MoSe_2_ PL intensity (photo counts in arbitrary units).

## Results

### Atomic Force Microscopy (AFM)

Nanosized protuberances were observed in the MoSe_2_ sample through AFM maps, with roughly circular shapes with radii ranging from 25 to 100 nm and heights ranging from 5 to 10 nm, as shown in Figure 2a,b. This region roughly matches the hyperspectral data set HS1. Protuberances are present in both the MoSe_2_ region and the hBN-only region, as shown in Figure 2e, which displays the coverslip/hBN and hBN/MoSe_2_ interfaces. The images were acquired in the tapping mode using a Park Systems XE-70 AFM.

**Figure 2:**
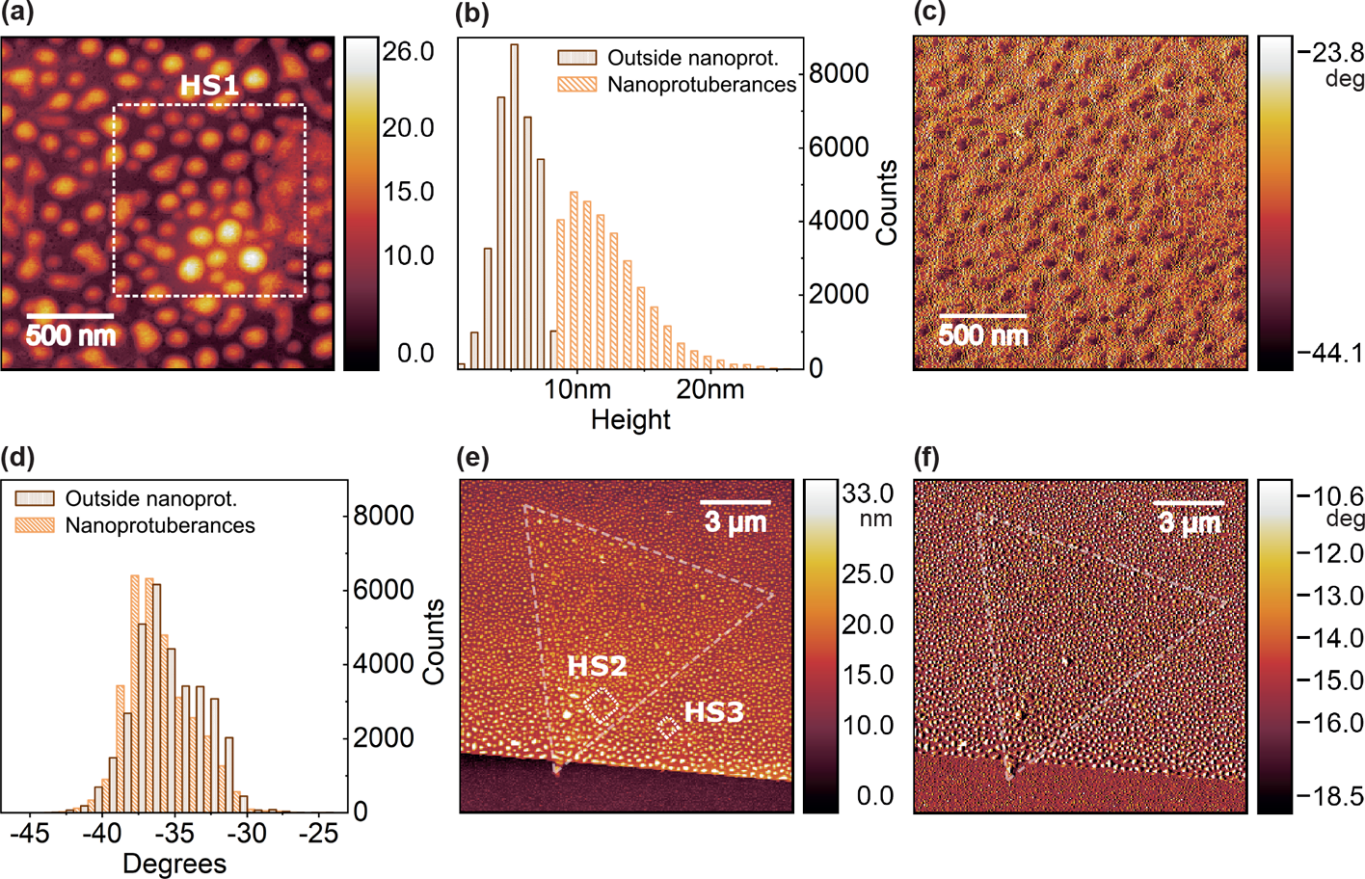
(a) Topographic map of the MoSe_2_ sample surface, revealing nanoscale protuberances measured by atomic force microscopy (AFM). The approximate location of the HS1 area is highlighted. (b) Histogram of height distribution, counted by pixels distinguished by regions inside and outside the nanoprotuberances. (c) Corresponding phase map. (d) Histogram of the phase contrast distribution. (e) Topographic map of the hBN edge with the MoSe_2_ flake outlined by dashed triangle lines, highlighting the approximate locations of the HS2 and HS3 regions. (f) Corresponding phase map, with the MoSe_2_ flake location similarly outlined by dashed lines.

In addition to topography, phase-contrast maps are acquired during tapping-mode AFM. Phase shifts indicate the difference between excitation and response of the cantilever and that contrast enables the differentiation of surface features at the nanoscale, indicating energy dissipation associated with tip–sample interactions.^23–25^ The maps in Figure 2c,f reveal phase contrasts when transitioning from protuberance regions to the surrounding areas. Additionally, a clearer contrast is observed between the coverslip and hBN, whereas the contrast at the hBN/MoSe_2_ interface is subtle. The histogram in Figure 2d indicates two distinct maxima in the phase distribution, distinguishing nanoprotuberance regions from flat areas. It is worth noting that the phase values are qualitative for the equipment used. By correlating the topography and phase contrast maps, the observed heterogeneous pattern can be attributed to the presence of contaminants between the material layers or on top of the MoSe_2_ flake.

Based on the AFM analysis, there are three possible scenarios to explain the presence of the nanoprotuberances in the topographic characteristics of the MoSe_2_ sample, as displayed in Figure 3: (a) imperfections in the MoSe_2_ layer depositing generating bubbles; (b) the presence of contamination trapped between MoSe_2_ and hBN; (c) the presence of contamination on top of the MoSe_2_ surface. Nano-Raman spectroscopy was utilized to explore these possibilities.

**Figure 3:**
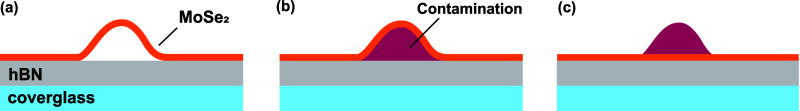
Schematic section representations (not to scale) of possible scenarios leading to the formation of nanoprotuberances in the MoSe_2_/hBN heterostructure on a coverglass. (a) Nanoprotuberances formed due to MoSe_2_ not adhering uniformly to the hBN surface. (b) Nanoprotuberance caused by contamination trapped between MoSe_2_ and hBN. (c) Contamination forming nanoprotuberances on top of the MoSe_2_ surface.

### TERS Figure of Merit for MoSe_2_ Contamination

Nano-Raman hyperspectral analyses were acquired from two distinct hyperspectral maps (HS1 and HS2) of the MoSe_2_ sample, as indicated in the APD images shown in Figure 1. On top of the nanoprotuberances, the intensity ratio of the *A*_1g_ band of MoSe_2_—240 cm^–1^—to the *E*_2g_ band—287 cm^–1^—distinguishes itself from flat regions across both maps acquired in MoSe_2_ regions (HS1 and HS2), as illustrated in Figure 4. This provides a figure of merit for spectrally differentiating regions inside and outside the protuberances based on the TERS hyperspectral data.

**Figure 4:**
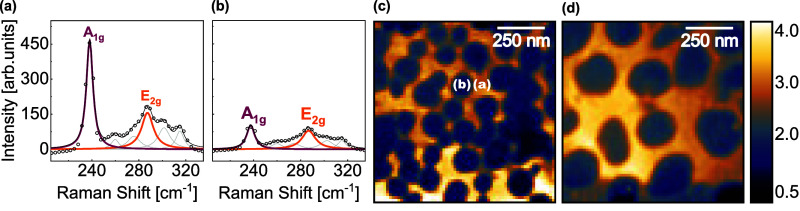
Comparison of MoSe_2_ characteristic TERS spectra (a) outside and (b) inside nanoprotuberances, corresponding respectively to regions (a) and (b) marked in (c). Intensity ratio of the MoSe_2_ *A*_1g_ band to the *E*_2g_ band mapped in (c) HS1 and (d) HS2. The color-coded scale bars represent intensity ratios (*I*_*A*_1g__/*I*_*E*_2g__).

In a far-field map acquired in the same region as HS1, the mean intensity ratio *I*_*A*_1g__/*I*_*E*_2g__ for the 4096 acquired spectra is (1.47 ± 0.08). A similar average analysis was performed for the two near-field maps, distinguishing between regions inside and outside the protuberances, an approach not feasible in the far-field due to the inability to spatially resolve regions of protuberance. Data were masked to separately analyze protuberances and their surroundings, given that in the two maps, areas of protuberance correspond to approximately 50% of the total mapped region. The mean intensity ratios are shown in Table 1.

**Table 1:** Mean TERS Intensity Ratios (*I*_*A*_1g__/*I*_*E*_2g__) Inside and Outside the Nanoprotuberances in HS1 and HS2

**map**	**nanoprotuberances**	**surroundings**
HS1	(1.5 ± 0.4)	(2.8 ± 0.8)
HS2	(1.7 ± 0.3)	(2.5 ± 0.5)

For protuberant areas, the mean intensity ratios found were (1.5 ± 0.4) and (1.7 ± 0.3) for hyperspectral maps HS1 and HS2, respectively, consistent with the mean intensity ratio observed in the far-field regime. In contrast, for regions outside the protuberances, the corresponding values were (2.8 ± 0.8) and (2.5 ± 0.5). These findings indicate that the strong enhancement of the *A*_1g_ mode observed in the near-field regime outside nanoprotuberances does not propagate to the far-field. This lower enhancement of the *A*_1g_ mode in the nanoprotuberances (when compared to the *E*_2g_ mode) is attributed to the phonon mode symmetry, which has implications in two different effects: (I) light coherence in the near-field and (II) polarization-dependent response. These two effects are discussed below.

(I) Coherence in Raman scattering refers to the preservation of phase correlations between the scattered fields originating from different regions of the sample, provided that the distance between these points is smaller than the phonon coherence length. This correlation is accessible only in the near-field regime, once the coherence length is limited to subwavelength scales, and whether it generates constructive or destructive field interferences depends on the phonon symmetry, as established for graphene.^26,27^ In graphene, the spectral features typically used to assess and quantify defects in far-field measurements—such as the *I*_*A*_1g__/*I*_*E*_2g__ intensity ratio^28^—are no longer directly applicable when TERS is employed. To address this discrepancy, the authors in^18,29^ developed a theoretical model based on spatial coherence,^26^ enabling defect analysis in near-field measurements using eq 1.

$${\left(\frac{A_{A_{1g}}}{A_{E_{2g}}}\right)}^{*}=\frac{A_{A_{1g}}^{*}}{1+\alpha [\sqrt{1-\beta (1-A_{A_{1g}}^{*})}-1]}-\gamma$$ 1

In this expression, *A*_*A*_1g__ and *A*_*E*_2g__ represent the peak areas of the corresponding vibrational modes, with the asterisk denoting near-field measurements normalized by far-field averages. The parameters α and β are related to the field enhancement factor (*f*_e_). The parameter γ varies according to the density of defects.

In Figure 5a, three distinct regions of the sample are identified, corresponding to measurements taken from nanoprotuberances, interfaces, and areas outside of the nanoprotuberances. Figure 5b shows a plot of the normalized area ratio between the *A*_1g_ and *E*_2g_ peaks [(*A*_*A*_1g__/*A*_*E*_2g__)*] as a function of the normalized *A*_1g_ peak area [(*A*_1g_)*], following the approach used in references.^18,29^ It is evident that the data follow a curve fitted by eq 1. This fit was obtained with α = 1.70, β = 0.58, and γ = 0. Since (*A*_1g_/*E*_2g_)* → 1 as (*A*_1g_)* → 1, the contaminants breaking TERS coherence length are very dense, with distances much smaller than the tip apex radius defining the resolution.^18^ The parameter β matches the values reported in graphene,^18,29^ whereas α differs, i.e., it is specific to our sample. Some points approach zero for the nanoprotuberance areas, which might indicate signal vanishing (see Figure S3 in the Supporting Information).

**Figure 5:**
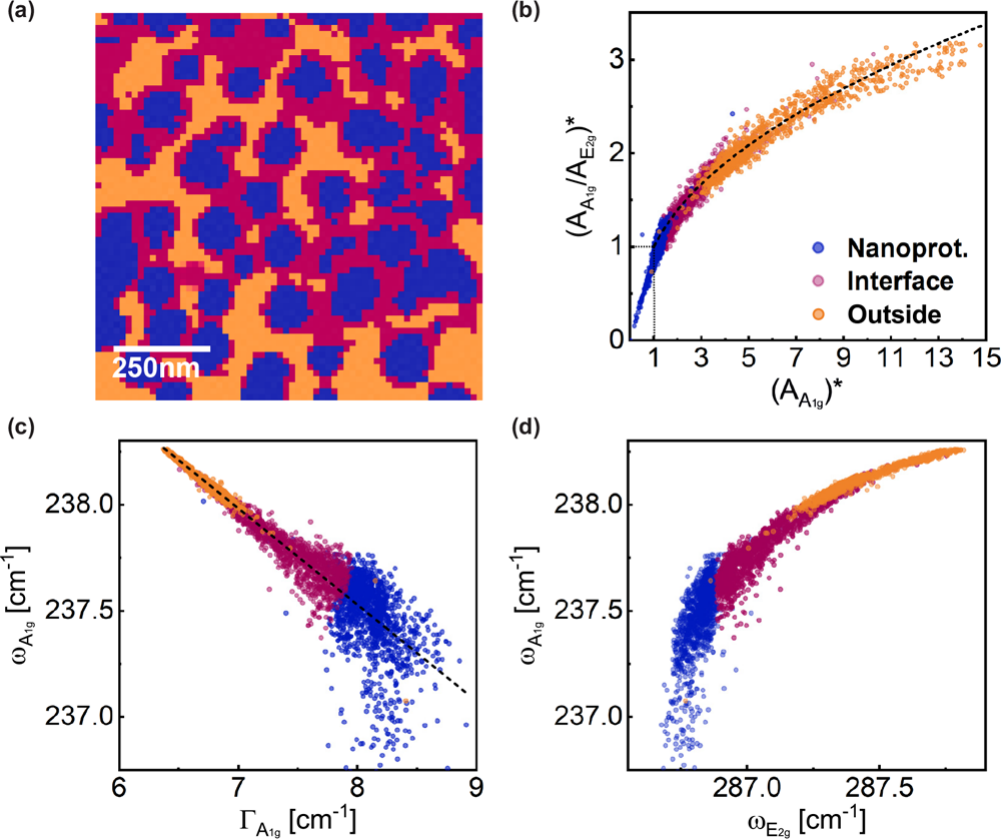
(a) HS1 map showing three regions of the sample, corresponding to measurements taken from nanoprotuberances, interfaces, and areas outside of the nanoprotuberances. (b) Normalized area ratio of the MoSe_2_ peaks (*A*_*A*_1g__/*A*_*E*_2g__)* as a function of the normalized area of the *A*_1g_ peak (*A*_1g_)*. (c) Position of the *A*_1g_ peak of MoSe_2_ as a function of the *A*_1g_ fwhm. (d) Position of *A*_1g_ as a function of the position of the *E*_2g_ peak of MoSe_2_.

The loss of coherence in the near-field on top of the nanoprotuberances indicates that the interaction length scale locally exceeds the phonon coherence length in these regions. This loss of coherence has two possible explanations: (I.i) a slight increase in tip–MoSe_2_ separation caused by the presence of contaminations on top of the MoSe_2_ flake or (I.ii) the scattering due to the presence of contaminations that decreases the TERS coherence length. Both mechanisms could contribute to the decrease in the *A*_1g_/*E*_2g_ ratio observed in the nanoprotuberances.

(II) In the near field, the enhancement of the electric field polarized perpendicular to the sample is considerably stronger than the enhancement of the field polarized within the sample plane. This effect should cause a higher enhancement in the *A*_1g_, since it has a non-null α_*zz*_^*A*_1g_^ Raman tensor, while α_*zz*_^*E*_2g_^ = 0. This effect has been neglected in TERS on graphene,^26,27^ where the Raman tensor α_*zz*_^*A*_1g_^ has been considered negligible. However, the same should not be valid for TMDCs,^30,31^ where the *A*_1g_-mode atomic displacements are perpendicular to the sample surface.^32^ In this scenario, the loss of selective *A*_1g_-mode enhancement due to stronger Z-polarized near-field on top of the nanoprotuberances can again be attributed to two possible explanations: (II.i) a slight increase in tip–MoSe_2_ separation caused by the presence of contaminations on top of the MoSe_2_ flake; (II.ii) local symmetry breaking due to the presence of contaminations causing lattice deformation or doping, reducing the near-field enhancement of the *A*_1g_ mode.

Finally, the observed values of the *A*_1g_/*E*_2g_ ratio at the interface between regions inside and outside the nanoprotuberances are due to the transition between these two areas, which may be either abrupt or gradual, depending on the TERS resolution dictated by the tip apex radius, on the order of 30 nm.

### Strain versus Doping in MoSe_2_

Figure 5c shows a plot of the *A*_1g_ mode frequency (ω_*A*_1g__) as a function of its full-width at half-maximum (fwhm Γ_*A*_1g__). The data points exhibit a linear trend, with lower frequencies and larger width values corresponding to the nanoprotuberance regions. A linear fit to the data presented in Figure 5c based on a linear expression (ω = *A*Γ + *B*) yields the parameters *A* = (−0.454 ± 0.003) and *B* = (241.16 ± 0.02) cm^–1^. Data points originating from the nanoprotuberances show greater dispersion from the linear trend compared with those from the surrounding regions. Points excluded from this analysis are discussed in the Supporting Information.

This behavior can be associated with doping, which is more pronounced in the nanoprotuberances. Both ω_*A*_1g__ and Γ_*A*_1g__ are known to be influenced by doping and can be expressed as linear functions of the doping level *d*, given by ω_*A*_1g__ = *a*_1_ *d* + *a*_2_ and Γ_*A*_1g__ = *a*_3_ *d* + *a*_4_, where *a*_1_, *a*_2_, *a*_3_, and *a*_4_ are constants. Isolating *d* in both equations and substituting one into the other leads to *A* = *a*_1_/*a*_3_ and *B* = *a*_2_ – (*a*_1_ *a*_4_)/*a*_3_. It is well established that n-type doping in TMDCs leads to a redshift and broadening of the *A*_1g_ Raman mode.^33^ Since the redshift is proportional to the electron concentration, this suggests that the nanoprotuberance regions contain a higher density of electrons. Although the *A*_1g_ mode is generally less sensitive to strain, it can also exhibit redshift and broadening under localized, nonuniform strain^18,34^ due to out-of-plane lattice deformation.

Besides the shift in the *A*_1g_ mode, the analysis also reveals a subtle shift in the *E*_2g_ mode, as seen in Figure 5d. Strain is known to have a significant impact on the frequency of the *E*_2g_ mode, which involves in-plane vibrations, typically causing a redshift accompanied by possible broadening or even spectral splitting if the symmetry is broken.^22,33,35,36^ As shown in Figure 5d, the *E*_2g_ mode exhibits a more pronounced redshift in the nanoprotuberances and at their edges, with an average redshift of approximately 1 cm^–1^. These spectral shifts may indicate the combined effects of strain and doping.

In addition to the analysis of Raman modes, PL spectroscopy serves as a valuable tool for probing electronic and structural changes.^37,38^ However, no significant shifts in the PL peaks were observed when comparing the nanoprotuberance PL spectra with flat region spectra.

The nanoprotuberances exhibit an average height of approximately *h* = 10 *nm* and an average radius of *r* = 50 *nm*. The strain ϵ can be estimated from these dimensions using the relation ϵ ≈ (*h*/*r*)^2^,^39^ resulting in an average strain of about 2%. A strain of this magnitude would lead to an energy shift in the PL peak on the order of tens of meV and would produce a more pronounced redshift in the *E*_2g_ mode compared to the *A*_1g_ mode,^40^ while doping typically induces a stronger redshift in the *A*_1g_ mode.^33^ However, the maximum shifts observed are Δω_*A*_1g__ = – 1.6 cm^–1^ for the *A*_1g_ mode and Δω_*E*_2g__ = −1.1 cm^–1^ for the *E*_2g_ mode, and the absence of any shift in the PL spectrum compatible with ϵ ≈ 2 supports the hypothesis that doping, rather than strain, is the dominant effect. This interpretation is in agreement with the scenario illustrated in Figure 3c. The difficulty in disentangling the effects of strain and doping is a recognized issue, and near-field measurements, despite their high resolution, always record the tip–sample interaction signal, which requires a comprehensive interpretation.^41,42^

### Chemical Analysis of Contaminants

In addition to MoSe_2_ characteristic modes, distinct Raman peaks were observed in the regions of protuberances, indicating chemical species unique to these areas, as listed in Table 2. In Figure 6, a characteristic peak related to the presence of protuberances is observed at 974 cm^–1^. When the amplitude of the Lorentzian fit corresponding to this peak is mapped, the nanoprotuberances become prominent as the amplitude is higher in these regions. In contrast, the Lorentzian associated with the nearby 998 cm^–1^ peak exhibits an inverse spatial distribution, with higher intensity in the regions surrounding the protuberances. The 998 cm^–1^ peak is likely associated with MoO_3_,^43–45^ suggesting possible oxidation of MoSe_2_ across the sample. The presence of the lower-frequency peak within the protuberances, close to the known vibrational frequency of MoO_3_, indicates local charge doping effects in these regions. MoO_3_ domains are known to act as defects in MoSe_2_, potentially inducing local doping effects, and their formation is commonly associated with exposure to ambient air, particularly at edges or defect sites where oxidation is more likely to occur.^44^

**Figure 6:**
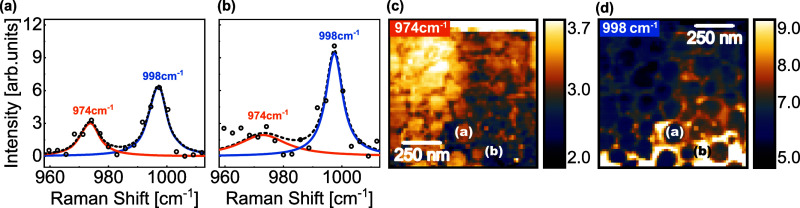
Curve fittings for characteristic peaks in (a) nanoprotuberance and (b) the region outside nanoprotuberances for the HS1 area. The orange Lorentzian corresponds to the peak at 974 cm^–1^, and the blue corresponds to 998 cm^–1^. Experimental data are represented by open black circles, while the dashed line corresponds to the final fitted curve. TERS intensity maps for (c) 974 cm^–1^ and (d) 998 cm^–1^ peaks, with highlighted pixels indicating the locations from which spectra (a) and (b) were extracted.

**Table 2:** Assignments for Distinct Raman Peaks Observed in Regions of Nanoprotuberances and Their Surroundings^a^

	**peak position (cm** ^**–1**^ **)**	
**assignment**	**nanoprotuberances**	**surroundings**
MoO_3_	974*/998	974/998*
C–C	1168*/1177	-/1177*
hBN	1365	1365*
O_2_	1555*	-
C=C	1579*/1587*/1600	1579/1587/1600*

a Asterisks indicate the regions where the peaks exhibit a maximum intensity.

Another example is presented in Figure 7, where three Lorentzian fits are shown, along with their corresponding amplitude distributions across the MoSe_2_ map. The most intense peak, located at 1587 cm^–1^, can be attributed to the C=C stretching mode, suggesting the presence of carbon-based contamination.^28^ The spatial distribution of the higher-frequency Lorentzian highlights regions surrounding the nanoprotuberances, whereas the lower-frequency component is localized primarily on the nanoprotuberances. This complementary spatial behavior resembles the pattern observed in Figure 6, indicating a common doping mechanism, stronger in the protuberance regions. A peak near the known vibrational frequency of molecular oxygen, at 1555 cm^–1^, was also detected in the nanoprotuberances. Although these features are very weak, they may suggest the presence of trapped gas in the nanoprotuberance regions. The corresponding data are presented in the Supporting Information.

**Figure 7:**
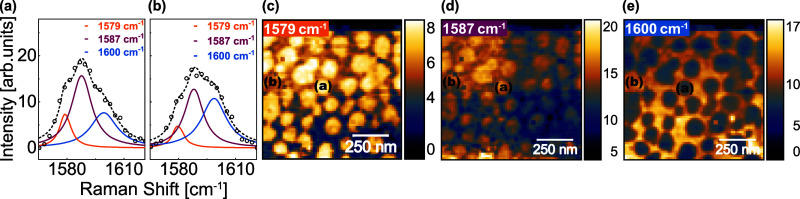
Curve fittings for characteristic peaks in (a) nanoprotuberance and (b) the region outside nanoprotuberances for the HS1 area. The orange Lorentzian corresponds to the peak at 1579 cm^–1^, the magenta corresponds to 1587 cm^–1^, and the blue corresponds to 1600 cm^–1^. Experimental data are represented by open black circles, while the dashed line corresponds to the final fitted curve. TERS intensity maps for (c) 1579 cm^–1^, (d) 1587 cm^–1^, and (e) 1600 cm^–1^ peaks, with highlighted pixels indicating the locations from which the spectra (a) and (b) were extracted.

Hyperspectral data from HS2 reveal that the nanoprotuberances are most prominent when the peak at 1168 cm^–1^ is selected, which is likely associated with vibrational modes of organic compounds, such as C–C bonds. A detailed analysis of this data set is provided in Supporting Information, along with complementary data from HS3, which corresponds to a region composed solely of hBN. The Raman peaks listed in Table 2 indicate the presence of MoSe_2_ oxidation products and other vibrational modes of organic compounds.

The origin of the oxidation in MoSe_2_ can be attributed to several factors. Exposure to ambient air or to transfer processes involving alkaline solutions is known to promote oxidation in TMDCs.^44^ Additionally, slower and more gradual oxidation processes under natural conditions tend to generate more stable oxides, which can progressively spread across the sample surface.^46^ Since oxidation is favored in regions with enhanced chemical reactivity, this may explain the higher concentration of MoO_3_ near the nanoprotuberances area. Nevertheless, oxidation is not confined to these areas and is observed across the entire sample, as shown in Figure 6. It is important to emphasize that oxide formation is an intrinsic transformation pathway of TMDC materials when exposed to environmental conditions.

### Physical-Chemistry Structure of the Nanoprotuberances

Given that oxidation is present throughout the sample, contamination is proposed as the main cause of the formation of these nanoscale features. Several possible mechanisms may explain the origin of the contamination-induced nanoprotuberances.

One hypothesis involves substrate roughness, where regions of MoSe_2_ may not establish full contact with the hBN, or where the hBN itself may not adhere perfectly to the cover glass, resulting in the formation of bubbles and folds,^47^ as illustrated in Figure 3a. This scenario is unlikely in the present sample, as the nanoprotuberances are distributed uniformly across the surface, and they exhibit Raman peaks related to different chemical species (see Table 2).

Another possibility is the presence of transfer residues from the polymer stamp used during fabrication.^47,48^ In this work, the sample was prepared using an Elvacite-based stamp, and none of its characteristic Raman peaks coincide with the contamination peaks observed in the sample. Moreover, the Elvacite residues are effectively removed through chloroform cleaning during fabrication, further weakening this hypothesis.

It is also important to rule out the hypothesis that the TERS tip picked up contamination from the nanoprotuberances along the measurements. In this case, the same spectral signatures could be seen everywhere. Some peaks are observed exclusively in the nanoprotuberance regions, while others are detected throughout the entire MoSe_2_ sample, as summarized in Table 2. Most of these peaks are absent in the HS3 region, which consists solely of hBN (see Supporting Information). The C=C-related peak is the only one present across all regions, including HS3, that was acquired after HS2 using the same TERS tip. The presence of this specific peak is likely not related to tip contamination, although this possibility cannot be ruled out with certainty.^44^

A more plausible explanation involves hydrocarbon contamination introduced during the fabrication process, where volatile organic species may become trapped between the MoSe_2_ and hBN layers^22,47^ or adhere to the sample surface through van der Waals interactions,^46^ as illustrated in Figure 3b,c. In Figure 3b, the contamination is trapped between the hBN and the MoSe_2_ layer, whereas in (c), it is located on top of the MoSe_2_. This scenario is further supported by the multiple heating and cooling steps involved in the sample preparation, which can promote the aggregation of hydrocarbon-based contaminants.^46^ Thermal cycling may lead to the formation of microscopic droplets containing hydrocarbons or other volatile species,^39^ which, upon drying, could aggregate into the observed nanoprotuberances. This hypothesis is particularly consistent with the fabrication, transport, and storage conditions of the sample prior to the measurements, and it is also compatible with the formation of oxidized regions mostly at the nanoprotuberances across the sample. Considering this hypothesis, together with the previously discussed effects—such as doping signatures, chemical analysis, and the possible mechanisms underlying the figure of merit—the scenario illustrated in Figure 3c stands out as the most plausible physical-chemistry structure for the nanoprotuberances.

## Conclusions

Nano-Raman and AFM analyses were conducted on MoSe_2_ exhibiting nanoprotuberances. A figure of merit was established to identify the presence of surface contaminants, based on the intensity ratio between characteristic MoSe_2_ Raman modes (*A*_1g_/*E*_2g_). A reduction in this ratio serves as a reliable nano-Raman signature of nanoprotuberances in monolayer MoSe_2_ samples. The *A*_1g_/*E*_2g_ changes are accompanied by changes in the *A*_1g_ and *E*_2g_ peak frequencies and line widths, and quantitative analysis is more aligned with local doping, although local strain cannot be ruled out. In addition, our nano-Raman data revealed spectral features associated with oxidation (MoO_3_) and contamination (organic compounds) within nanoprotuberances, with further indications of a stronger charge transfer between MoSe_2_ and contamination species located at the protuberances. These results contribute to a more comprehensive understanding of the nanoscale surface chemistry and degradation processes in two-dimensional materials.

## Methods

The sample was prepared by using a dry-stamping approach. Initially, an hBN flake was lifted with a polymer stamp. The MoSe_2_ grains were grown on a SiO_2_/Si substrate by salt-assisted chemical vapor deposition (CVD) and then picked up by hBN flakes (MoSe_2_/hBN), as described previously.^47^ The individual MoSe_2_ layers were sequentially lifted and rotated relative to each other during the stacking process, ensuring the formation of well-defined mono- and bilayer regions only. Finally, the assembled structure was transferred to a glass slide using another polymer stamp, ensuring proper layer alignment while avoiding direct contact with the SiO_2_ substrate (coverglass).^47,49^ In this context, hBN serves as an atomically flat surface that minimizes wrinkles, which improves the quality of the MoSe_2_ layers, while SiO_2_ acts as the substrate for the final support of the heterostructure.

For this nano-Raman spectroscopy study, we employed TERS based on AFM to analyze the sample. TERS measurements were performed using the Porto Laboratory prototype system, which operates in bottom illumination mode with a noncontact AFM setup utilizing a tuning fork.^50^ The system is equipped with a He–Ne radially polarized as the excitation source, an avalanche photodiode (APD) detector and an Andor Shamrock 303i spectrometer with a 600 l/mm grating. The TERS probes used in this experiment were PTTP (Plasmon Tunable Tip Pyramids) probes, specifically chosen for their ability to enhance Raman scattering through localized surface plasmon resonance.^51–53^ The nano-Raman hyperspectra were recorded with a step length of 15.6 nm. The HS1 region was analyzed using a tip that provided a 25-fold enhancement for the *A*_1g_ MoSe_2_ mode (tip in/tip out contrast), while the remaining two (HS2 and HS3) were analyzed with a tip that offered a 10-fold spectral enhancement for the same Raman peak.

The acquired data was processed using PortoFlow Analysis software, where principal component analysis (PCA) was applied to improve the data quality. PCA transforms the data into a lower-dimensional space composed of only five principal components as reconstructed data, highlighting the most relevant information and enabling the identification of key features in the Raman spectra. This approach improved the clarity of the spectral data, particularly in visualizing the intensity and spatial distribution of weak Raman peaks across the sample. The spectral regions of interest were selected, and the background was removed to allow proper curve fitting. This procedure produced intensity maps based on the properties of the Lorentzian peaks used to fit the spectral features.

For completeness, AFM measurements were performed using a Park Systems XE-70 AFM operating in tapping mode with lateral resolution close to 5 nm. The AFM analysis was crucial for providing AFM-standard topographical information on the sample, allowing us to correlate structural features with the Raman data obtained from the TERS measurements.

## Supplementary Material



## Notes

Monolayer PL measurements; far-field measurements; extended plots of Figure 5 (MoSe_2_ modes properties); detailed chemical analysis of contaminants, including zoomed views of nanoprotuberances and characterization of the 1555, 1168, 1177, 1226, and 1423 cm^–1^ peaks; and Raman spectrum of the polymer stamp (PDF)
