# Supplementary material for: Nano-Raman Spectroscopy Figure of Merit and Chemical Analysis of Contaminations in Single-Layer MoSe2
Source: ACS Nano. 2025 Sep 11;19(40):35438–46. doi: 10.1021/acsnano.5c08036 (PMC12530041; doi:10.1021/acsnano.5c08036)
Supplement: Supplementary file 1 [file nn5c08036_si_001.pdf]

Supporting Information for: Nano-Raman Spectroscopy Figure of Merit and Chemical Analysis of Contaminations in Single-Layer $MoSe_2$

Jane Elisa Guimarães,[†] Rafael Nadas,[‡] Rayan Alves,[†] Wenjin Zhang,[¶] Takahiko Endo,[¶] Kenji Watanabe,[§] Takashi Taniguchi,[§] Riichiro Saito,[¶,‖] Yasumitsu Miyata,[¶] Bernardo R. A. Neves,[†] and Ado Jorio[*,†]

†*Departamento de Física, Universidade Federal de Minas Gerais, Belo Horizonte, MG, 31270-901, Brazil*

‡*Institut fur Physik, Humboldt-Universitat zu Berlin, Newtonstraße 15, 12489, Berlin, Germany*

¶*Department of Physics, Tokyo Metropolitan University, Tokyo, Japan*

§*National Institute of Materials Science (NIMS), Tsukuba, 305 0044, Japan*

‖*Department of Physics, Tohoku University, Sendai, Japan*

E-mail: adojorio@fisica.ufmg.br

S1. Monolayer PL measurements

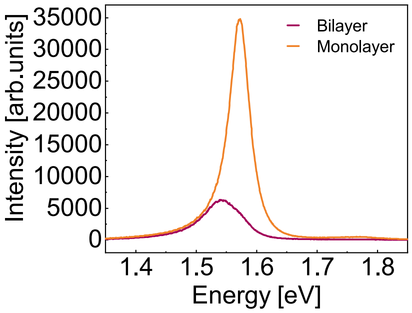

Figure S1: Confocal photoluminescence (PL) spectra acquired from two distinct regions of the $MoSe_2$ sample: monolayer and bilayer.

The $MoSe_2$ monolayer exhibits a pronounced photoluminescence (PL) peak at 1.57 eV, corresponding to the direct A exciton [37] as seen in Figure S1. In contrast, the bilayer region displays a PL peak at 1.54 eV, with an intensity approximately 5.5 times lower than that of the monolayer. The avalanche photodiode (APD) used is equipped with a bandpass filter centered at (760 ± 12) nm, enabling selective detection of the PL signal associated with the monolayer emission.

S2. Far Field measurement

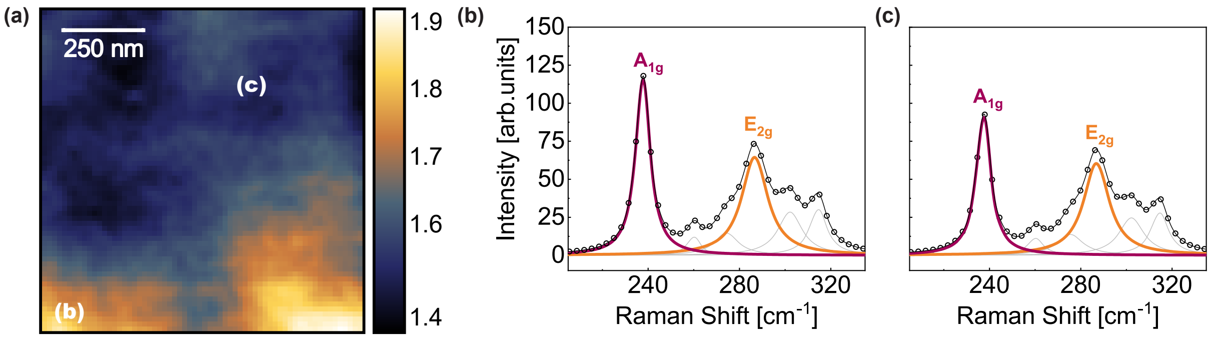

Figure S2: (a) Intensity ratio map of the A_{1g} to E_{2g} Raman bands of $MoSe_2$ for HS1 in the far-field regime. Representative spectra are shown at the pixel locations highlighted in (b) and (c). The color-coded scale bar represents the intensity ratio, ranging from lower values (blue regions) to higher values (orange regions). (b) Spectrum from the map corresponding to a ratio of 1.8. (c) Spectrum from the map corresponding to a ratio of 1.5. Experimental data are shown as open black circles.

S3. $MoSe_2$ modes properties

Figures S3 (a) and (b) show the points not presented in Figure 5 (c) and (d) in the main text. The points that deviate from the trend line are associated with regions of noisy hyperspectral data, and are indicated in white in the inset in Figure S3(b). In (c), the intensity ratio ($I_{A_{1g}}/I_{E_{2g}}$) is plotted as a function of the full width at half maximum (Γ) of the A_{1g} mode. In this case, the ratio decreases as Γ increases. The points falling outside the main trend in (a) also deviate from the main trend here, with low intensity of A_{1g}. Figure S3(d) presents the frequency of the E_{2g} mode as a function of its FWHM. A redshift of $\Delta\omega_{E_{2g}} = -1.1\,cm^{-1}$ is observed for this mode at the nanoprotuberances.

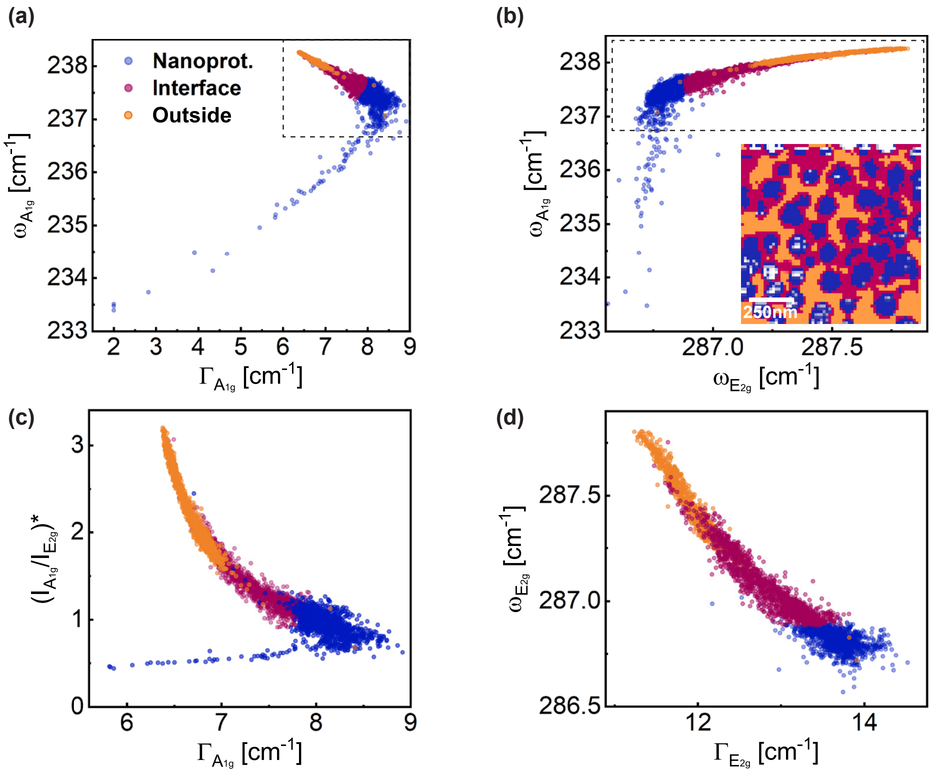

Figure S3: (a) Extended plot of the position of the A_{1g} peak of $MoSe_2$ as a function of A_{1g} FWHM. The region shown in the upper-right corresponds to the area displayed in Figure 5(c). (b) Extended plot of the position of the A_{1g} mode as a function of the position of the E_{2g} mode. The inset shows the same map as in Figure 5(a), with white pixels indicating the points that fall outside the main trend in Figure 5 (c-d). (c) Normalized intensity ratio of the $MoSe_2$ modes as a function of A_{1g} FWHM. (d) Position of the E_{2g} peak as a function of E_{2g} FWHM.

S4. Chemical analysis of contaminants

Figure S4 provides a more detailed analysis of the HS1 region. In Figure S4(a), the map of the E_{2g} peak position of $MoSe_2$ reveals a redshift of this vibrational mode in the areas corresponding to the nanoprotuberances. The zoomed-in view in Figure S4(b) shows that the peak position exhibits a gradient that follows the topographical profile. Figure S4(c) presents the same zoomed-in region, but now showing the intensity map of the 998 cm^{-1} peak, corresponding to the map in Figure 6(d) of the main text. A similar gradient is observed, with the intensity of this peak progressively decreasing towards the center of the

nanoprotuberance. Figure S4(d) displays the intensity map of the 1579 cm^{-1} peak, as shown in Figure 7(c). The observed gradients fall within the spatial resolution limits of the TERS system used.

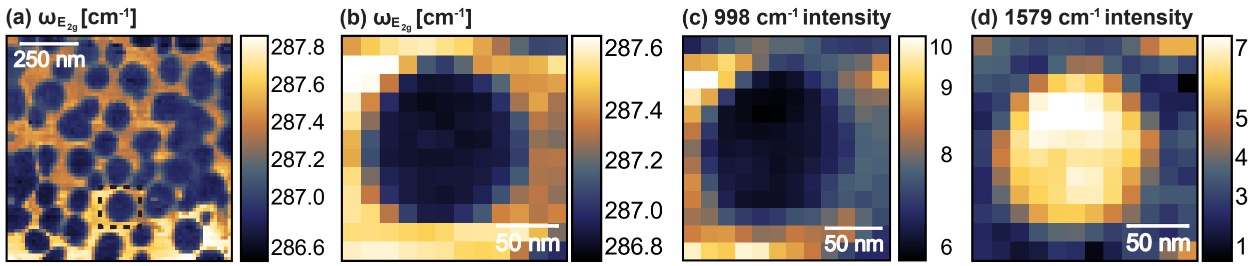

Figure S4: (a) Spatial distribution of $\omega_{E_{2g}}$ (peak position in cm^{-1}) over the sample surface. The dashed black square indicates the area enlarged in (b-d). (b) Spatial distribution of $\omega_{E_{2g}}$ within the highlighted region. (c) Raman intensity map of the 998 cm^{-1} peak over the same area. (d) Intensity map of the 1579 cm^{-1} peak. All intensity values shown are in arbitrary units.

A weak peak around 1555 cm^{-1}, close to the characteristic vibrational frequency of gas-phase oxygen, was also detected in the nanoprotuberance regions, as shown in Figure S5. The spatial distribution of this peak intensity resembles that observed for the 974 cm^{-1} peak (Figure 6 in the main text), which is associated with oxidation.

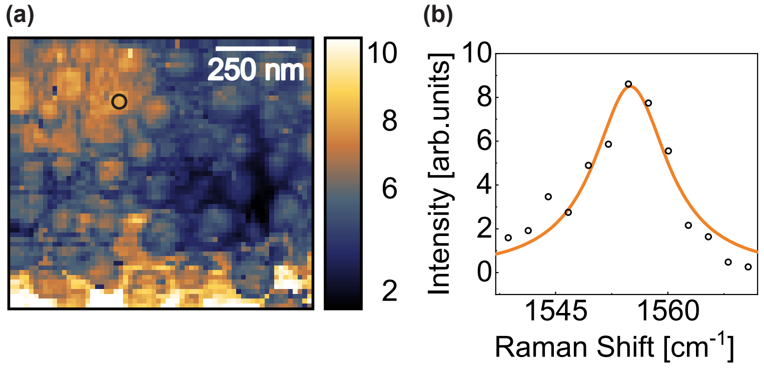

Figure S5: (a) Intensity TERS map of the 1555 cm^{-1} peak in HS1. (b) Corresponding Raman spectrum from the pixel indicated by the black circle in (a). Experimental data are shown as open black circles.

For HS2, the nanoprotuberances become prominent when selecting the peak at 1168 cm^{-1}, as shown in Figure S6. A similar pattern to that observed in HS1 is identified, with multiple

Lorentzian components contributing to the signal: the lower-frequency Lorentzian is more pronounced at the locations of the nanoprotuberances, while the higher-frequency component is more intense in the surrounding regions. The peak at 1168 cm^{-1} is likely attributed to vibrational modes of organic compounds, such as those involving C-C bonds, which may also indicate the presence of carbon contamination. The map shown in Figure S6 was acquired at the edge of the $MoSe_2$ flake, which accounts for the presence of the observed peak in regions that would otherwise be expected to be flat. It might be due to irregular adhesion of the flake to the underlying hBN or the accumulation of contaminants at the flake boundary at the bottom of the map.

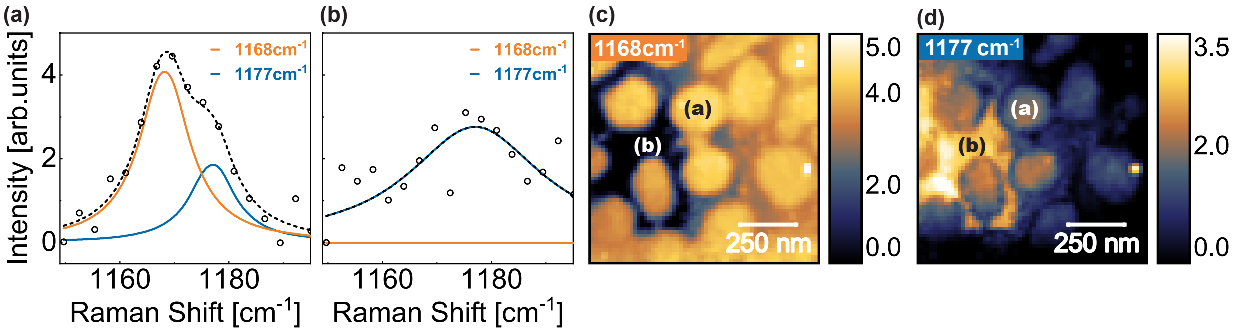

Figure S6: Curve fitting of characteristic peaks in (a) nanoprotuberance regions and (b) surrounding areas for the HS2. The orange Lorentzian represents the peak at 1168 cm^{-1}, while the blue corresponds to 1177 cm^{-1}. Experimental data are shown as open black circles, and the dashed line indicates the resulting fitted curve. TERS intensity maps for (c) 1168 cm^{-1} and (d) 1177 cm^{-1} peaks, with highlighted pixels indicating the locations from which the spectra (a) and (b) were extracted.

The HS3 hyperspectrum was collected from a region without the presence of $MoSe_2$, where nanoprotuberances are observed over hBN. The maps of the peaks in these regions reveal both similarities and differences compared to the peaks found in HS1 and HS2. In HS3, the peaks most prominently highlighting the nanoprotuberances in intensity maps are at 1226 and 1423 cm^{-1}, as shown in Figure S7. The first can be attributed to hydrogenated organic compounds (CH_2 or C-O-H), and 1423 cm^{-1} is associated with CH_2 vibration. A single Lorentzian was used to fit the data in these maps, and these peaks are not observed in $MoSe_2$ (HS1 and HS2).

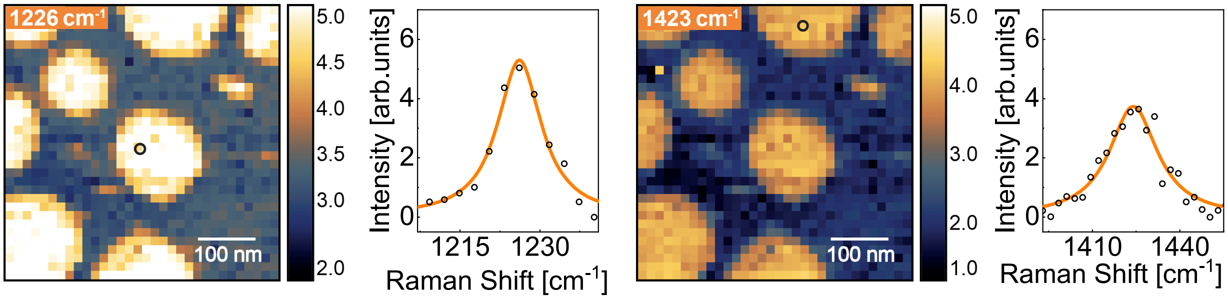

Figure S7: Intensity TERS maps of the 1226 cm^{-1} and 1423 cm^{-1} peaks in nanoprotuberances for HS3 and their corresponding curve fits. Experimental data are represented by open black circles. The black circle indicates the pixel in the map from which the spectrum data was extracted.

S5. Raman spectrum of the polymer stamp

The observed contamination is not related to the polymer stamp used in the fabrication process. In this work, the sample was prepared using a stamp made of Elvacite. For reference, the Raman spectrum of Elvacite is provided in Figure S8. None of the characteristic Raman peaks of Elvacite match the contamination peaks detected in the nanoprotuberances.

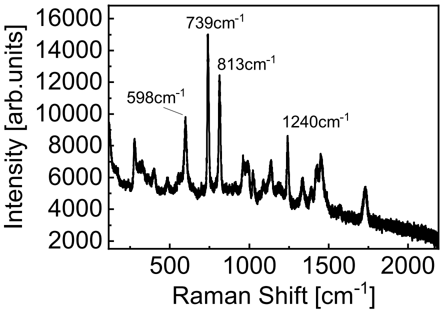

Figure S8: Raman spectrum of the Elvacite polymer stamp used in the fabrication process.
